# Monthly Summary

**Published:** 1872-02

**Authors:** 


### MONTHLY SUMMARY.

*Epulis.*—Maxie T., aged 7 years, has a tumor on the gum, which has existed for about three months. Last week he had a tooth extracted, which seemed to be intimately connected with the mass. This tumor is of a fibroid character, and undoubtedly commenced its growth in the socket of the tooth, which it loosened and pushed before it. The tumor was defined as an epulis, and is unusual at this period of life, the lecturer stating that he had never met with a growth of this kind at so early an age. Sometimes such a structure as this takes on epithelial action ; at present it is the seat of a discharge of a disagreeable odor. A tumor of this kind may acquire sufficient bulk to interfere with mastication, deglutition, etc. It is liable to return and the only remedy for its relief is excision of the piece of bone to which it is attached.

Chloroform was administered, and on taking firm hold of the growth it peeled off from the jaw like a piece of old moss from the bark of a tree. Two teeth that seemed implicated were drawn, and the jaw bone, being soft, was thoroughly chiselled out, instead of a section being removed by means of the saw.

The especial care that must be taken with patients while under the influence of an anæsthetic during operations about the mouth was well shown in this case. The parts were very vascular, and repeatedly the patient had to be turned on his side and the head depressed, in order to evacuate the blood, which constantly ran into his windpipe and threatened to strangle him.

In connection with this case, Prof. Gross exhibited to the class two pathological specimens, removed in private practice during the morning. The first and larger one was a growth taken from the right fore-arm of an old lady seventy-two years of age. It had the appearance of a mushroom, and the skin on which it rested was greatly congested. It discharged a thin and offensive sanious fluid, but was the seat of no positive pain, if a slight twinge now and then might be excepted. It rested on a broad base, but, on removal, the subcutaneous fatty and cellular tissues, to all appearances, were found to be perfectly healthy.

Section of it resembled section of the pancreas or mammary gland of a young subject. It occasionally bled, and was undoubtedly an epithelial outgrowth of the skin.

The second specimen resembled a large wart, and was taken from the lower lip of a man aged eighty years. Its base was greatly indurated, and it was defined as an epithelial cancer.—*Surgical Clinic of Prof. Gross.—Medical Times.*

---

*A Simple and Excellent Cleansing Agent.*.—Ammonia, or as it is more generally called, spirits of hartshorn, is a powerful alkali, and dissolves grease and dirt with great ease. It has lately been recommended very highly for many domestic purposes. For washing paint, put a teaspoonful in a quart of moderately hot water, dip in a flannel cloth, and with this simply wipe off the woodwork ; no scrubbing will be necessary. For aking grease spots from any fabric, use the ammonia nearly pure, then 'lay white blotting paper over the spot, and iron it lightly. In washing laces, put about twelve drops in a pint of warm suds. To clean silver, mix two teaspoonfuls of ammonia in a quart of hot soap suds. Put in your silverware and wash it, using an old nail-brush or tooth-brush for the purpose. For cleaning hair-brushes, etc., simply shake the brushes up and down in a mixture of one teaspoonful of ammonia to one pint of hot water ; when they are cleansed, rinse them in cold water, and stand them in the wind or in a hot place to dry. For washing finger marks from looking glasses or windows, put a few drops of ammonia on a moist rag, and make work of it. If you wish your house plants to flourish, put a few drops of the spirits in every pint of water used in watering. A teaspoonful in a basin of cold water will add much to the refreshing effect of a bath. Nothing is better than ammonia water for cleansing the hair. In every case rinse off the ammonia with clear water. To which we would only add, that for removing grease spots, a mixture of equal parts of ammonia and alcohol is better than alcohol alone ; and, for taking out the red stains produced by strong acids in blue and black clothes, there is nothing better than ammonia.—*Technologist.—Richmond and Louisville Med. Journal.*

*Infection—Ready Mode of Preventing.*—There is perhaps no plan of preventing infection so ready as the production of sulphurous acid by the combustion of sulphur. To disinfect a bed, whilst the patient is temporarily removed from it, pass a copper warming-pan into the bed, containing a few live embers and a little sulphur. The pan should be moved about during the ignition of the sulphur. By burning sulphur in an open vessel, closets, carriages, passages, and vacated chambers of the sick may be easily disinfected. Clothing may be lightly sponged over or sprinkled with water containing a little well mingled sulphur, and then ironed with a flat-iron heated to a temperature which will cause ventilation of the sulphur without burning the linen.—MR. J. SARTIN.—*Braithwaite.*

---

*Earache in Children.*—Dr. Anstie, after detailing (*The Practitioner*) the particulars of an attack of auricular herpes zoster in his own person, says that the idea has occurred to him that a large proportion of cases of earache, which are generally set down as examples of suppurative inflammation of the meatus auditorius, are in reality nothing but neuralgic herpes, depending on an affection of the auriculo temporal nerve. In the earache to which children are liable, the pain often comes on in the first instance violently; the patient cries out with the severity of the suffering, but then there are remissions, amounting often to complete intermissions. Moreover, it is but rarely that a well defined discharge of pus is observed, even when relief to the pain has been somewhat suddenly obtained, as is not unfrequently the case. On the contrary, there is frequently found a shedding of small scales, either dry or moistened with a little bloody sanies, from the lining of the meatus, a few days after ths cessation of the pain—in fact, what looks like the dead epidermis of vesicles that have faded. The whole course of events seems, he says, to correspond much more nearly with that of auricular neuralgic herpes than with that of common abscess of the lining membrane of the meatus. Herpes zoster occurring in children on other parts of the body is not attended by much neuralgic pain, and the occurrence of severe pain in the auricular form of the disease is difficult to explain. Dr. Anstie suggests that it may be due to the fact that the superior and inferier maxillary nerves are the first of all sensory nerves in the body to exhibit any tendency to pain of a grade approaching to the severity of true neuralgia ; and the reason of this is, possibly the depressing and exhausting influence exerted on them by the growth of the teeth.—*Medical Times.*

---

*Vegetable Carbolic Acid.*—We read that a plant called the andromeda leschenanltil, growing in the Neilgherry hills, in India, has been found to yield carbolic acid. Mr. Broughton, the Government medical officer for the district, reports that it is far superior in purity to the ordinary product of coal tar, being less deliquescent and free from any admixture of noxious concomitants. As its cost is far above that of themineral product, and as the latter can be chemically purified the discovery has no economical or commercial value ; but it is interesting as a botanical and chemical fact.—*Druggist Circular.*

---

*Fatal Salivation from Bichloride of Mercury.*—In a case which is fully reported in the *Lancet* for September 16th, Dr. Meeres applied with a small camel's hair brush a strong alcoholic solution of corrosive sublimate—eighty grains to the ounce—to the head of a child affected with tinea tonsurans. The application gave rise to no pain at the time, but during the ride home, in an open dog-cart, the child suffered severely. Shortly afterwards vomiting and purging came on. Salivation, accompanied by much swelling of the parotid and submaxillary glands, was first observed on the evening of the day after the application, and continued until death took place, apparently from prostration, on the morning of the fifth day.

The verdict of the coroner's jury was "that death was caused by poison from the application of a very strong preparation of bichloride of mercury made to the head and neck by Dr. Meeres," and that "Dr. Meeres is very greatly to be blamed for having made the application."

The lotion applied was from a formula of Dr. Tilbury Fox, and has been used by him in a precisely similar manner in the same disease in very many instances, and this case is the first in which any untoward symptoms have been produced by it.—*Medical Times.*

*A New Way of Testing Petroleum.*—At the session of the American Science Association in Indianapolis, Prof. Vander Weyde described his new test for the adulteration of kerosene, which seems to be of immediate practical value. This is founded on the fact that all vapors given off by petroleum are combustible, and that if any kerosene or other preparation from petroleum gives off a vapor at the accepted standard temperature of 110 degrees, and it is not necessary to try whether it will burn, but sufficient to collect it in a proper vessel, by which we gain the additional advantage that we may measure the quantity of the vapor, while none of it can be lost by air currents incidentally passing over the surface of the liquid. He takes, therefore, a glass tube, closed at one end and open at the other, and fills it with the petroleum to be tested, then, closing the open end with the finger, inverts it in a vessel with water warmed to 110 degrees by mixing hot and cold water, and keeping it at that temperature by occasionally adding hot water. If now any vapor be apparent, it will collect in the closed upper part of the tube, displacing the oil downward. The amount of this gas will be a comparative test of the different qualities of oil, and for this purpose the tube may be graduated in order to measure the amount of volatile liquid present in the same. This method is not subject to the discrepancies found in the usual way of testing, in which an impure and dangerous quality of oil may be made to appear better than it is by slow and gradual heating, and which if performed in a light draft of air will have the vapors carried off as soon as developed, so that it becomes impossible to ignite them. This new method gives freedom from the danger of using fire, more accuracy, a trustworthy means of measurement, and no chance for deception.—*Medical and Surgical Reporter.*

---

*Poisoned Gloves.*—An English medical journal publishes a warning against the wearing of green kid gloves. It has been observed in several cases that the hands of those wearing gloves of this color soon become covered with an eruption which physicians find hard to cure, as the poison seems to enter the system. Upon analysis, it has been found that the green used for dyeing the kid contains arsenic. Though not all the green kid gloves in the market are so dyed, it is nevertheless safer to wear others of a less bright and less dangerous color.—*Druggists' Circular.*

---

*Cotton to Stop Bleeding*—A hæmostatic can be prepared, according to Dr. Ehrle, by first digesting the raw material in caustic soda, and afterward saturating with chloride of iron. It has been thoroughly tried in the hospitals of Europe during the last six months, and is everywhere highly commended. The application of it is in the same way as lint, and as it absorbs water, close bottles or packages must be employed for preserving it. It would be a good article to have on hand in every household. We mentioned some time ago a modification of this process in the form of gun cotton, to be used as a disinfectant, by substituting permanganate of potash for the chloride of iron. As long as gun cotton is kept wet there is no danger of its taking fire, and the fiber in this condition is not liable to disintegrate by the action of the agents poured upon or through it. Gun cotton is one of the best agents we have for retaining liquids for any purpose.—*London Ph. Journal.*

---

*Death from Methylene*—A recent number of the *Oxford Chronicle* a fatal case in the Radcliffe Infirmary, Oxford. The patient, a married woman, agged 44, was about to undergo an operation for cancer of the breast. Bichloride of methylene was administered by the dispenser of the Infirmary, in the presence of the house-surgeon and one of the surgeons, on a flannel bag. After two or three convulsive gasps, the patient expired. The quantity administered was small. Artificial respiration was practiced, and other means of restoration, but without success.—*Brittish Medical Journal.*

---

*Uses of Carbolic Acid.*—In pasting wall papers, posters, etc., especially where successive layers are put on, there arises a most disagreeable effluvia, which is particularly noticeable in damp weather. The cause of this is the decomposition of the paste. In close rooms it is very unwholesome, and often the cause of disease. In large manufactories, where large quantities of paste is used, it often becomes sour and offensive. Glue, also, has often a very disagreeable odor. If, when making paste or glue, a small quantity of carbolic acid is added, it will keep sweet and free from offensive smells. A few drops added to mucillage or ink prevents mold. In whitewashing the cellar and dairy, if an ounce of carbolic acid is added to each gallon of wash, it will prevent mold and prevent the disagreeable taints often perceived in meats and milk from damp apartments.

Another great advantage in the use of carbolic acid in paste for wall paper, and in whitewash, is that it will drive away cockroaches and other insect pests. The cheapest and best form of carbolic acid is crystal, which dissolves in water or liquifies at an excess of temperature.—*Jour. Ap. Chemistry.*

